# Multienzyme Biosynthesis of Dihydroartemisinic Acid

**DOI:** 10.3390/molecules22091422

**Published:** 2017-08-28

**Authors:** Xixian Chen, Congqiang Zhang, Heng-Phon Too

**Affiliations:** 1 Biotransformation Innovation Platform, Agency for Science Technology and Research, Singapore 138673, Singapore; congqiang_zhang@biotrans.a-star.edu.sg; 2 Department of Biochemistry, National University of Singapore, Singapore 117598, Singapore

**Keywords:** whole cell biocatalysis, CYP71AV1, dihydroartemisinic acid

## Abstract

One-pot multienzyme biosynthesis is an attractive method for producing complex, chiral bioactive compounds. It is advantageous over step-by-step synthesis, as it simplifies the process, reduces costs and often leads to higher yield due to the synergistic effects of enzymatic reactions. In this study, dihydroartemisinic acid (DHAA) pathway enzymes were overexpressed in *Saccharomyces cerevisiae*, and whole-cell biotransformation of amorpha-4,11-diene (AD) to DHAA was demonstrated. The first oxidation step by cytochrome P450 (CYP71AV1) is the main rate-limiting step, and a series of N-terminal truncation and transcriptional tuning improved the enzymatic activity. With the co-expression of artemisinic aldehyde dehydrogenase (ALDH1), which recycles NADPH, a significant 8-fold enhancement of DHAA production was observed. Subsequently, abiotic conditions were optimized to further enhance the productivity of the whole-cell biocatalysts. Collectively, approximately 230 mg/L DHAA was produced by the multi-step whole-cell reaction, a ~50% conversion from AD. This study illustrates the feasibility of producing bioactive compounds by in vitro one-pot multienzyme reactions.

## 1. Introduction

Terpenoids are an important class of natural products that play a critical role in many industrial sectors, such as healthcare, flavor and fragrance, and biofuels [1,2]. Biochemically, they are synthesized from the two C5 molecules, namely isopentenyl pyrophosphate (IPP) and dimethylallyl pyrophosphate (DMAPP). Their structural diversity is the result of the terpene synthase reaction and stereospecific hydroxylation reaction by cytochrome P450 enzymes [3]. P450 monooxygenase is a heme-containing enzyme that actives a saturated carbon by inserting an oxygen atom [4,5]; hence, it provides a synthetic handle on the terpene skeleton for further modification, such as glycosylation or esterification [6].

Although many P450 sequences have been elucidated, very few have been successfully applied in heterologous hosts for the production of oxygenated terpenoids [6,7]. The challenges in using these enzymes include poor protein expression [7], inhibition of intermediate metabolites, and poor substrate-enzyme interactions [8]. Recently, Biggs and co-workers reported extensive engineering of P450 enzyme in *Escherichia coli* (*E. coli*), and achieved ~570 mg/L of oxygenated taxanes [9]. They noted that when the P450 enzyme was co-expressed with the upstream diterpene biosynthetic pathway, a measurable reduction in titer was observed, suggesting that the intracellular resources might be unduly perturbed [9]. Although balancing pathways in vivo has been extensively studied [10,11], it is still largely a combinatorial approach that requires significant amount of effort to construct and screen for the best producer strain. Moreover, to produce the final product, for example taxol, more than one P450 monooxygenase may be required [12], which makes in vivo pathway engineering a daunting task. Engineering microbial consortium is an attractive way to modularize the long metabolic pathways into different microbial hosts [13]. However, it is still restricted by the intrinsic properties of microorganisms. Using resting cells for whole-cell biocatalysis is an attractive, alternative strategy, which offers flexibility in tuning the reaction pH, temperature and media compositions that may not be tolerable for microorganisms growth [14]. Furthermore, multienzymes can be encapsulated inside the cells and separated from the reaction mixture easily for downstream processing.

**Figure 1:**
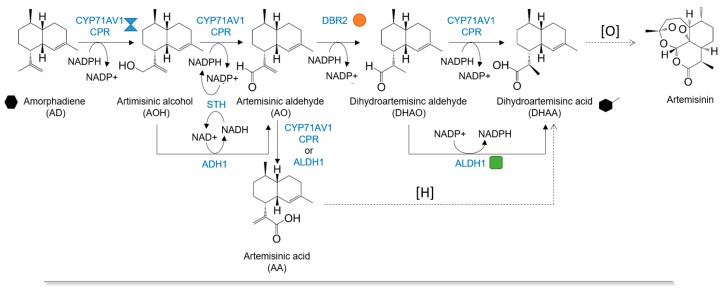

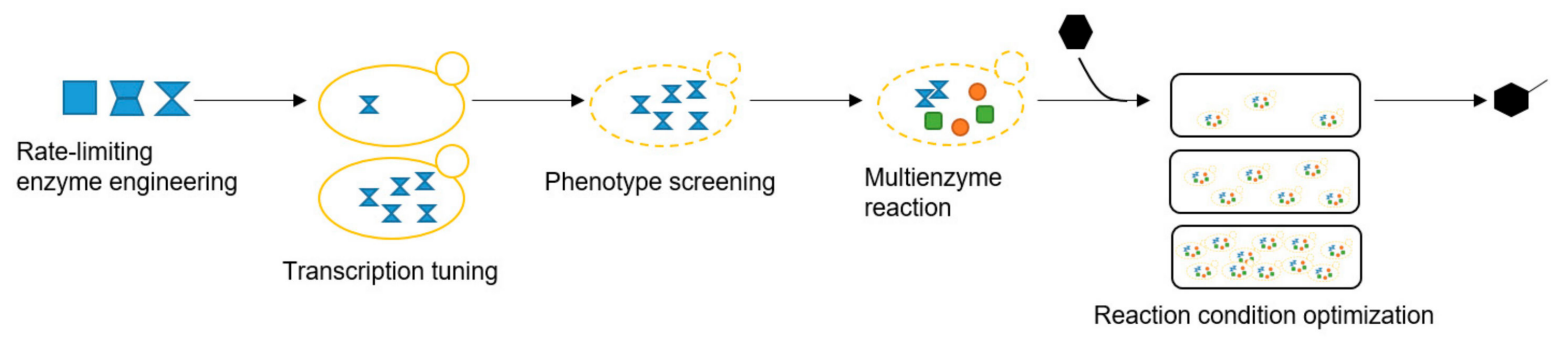
The schematic REPRESENTATION of the dihydroartemisinic acid synthesis pathway from amorphadiene and the optimization workflow for the yeast whole-cell biocatalysis. The abbreviations for the metabolites were shown in parentheses. The enzyme names are as follows: CYP71AV1, cytochrome P450 monooxygenase from *Artemisia annua*; CPR, cytochrome P450 reductase from *Artemisia annua*; DBR2, artemisinic aldehyde Δ11(13) reductase from *Artemisia annua*; STH, transhydrogenase from *Escherichia coli*; ADH1, artemisinic alcohol dehydrogenase from *Artemisia annua*; ALDH1, artemisinic aldehyde dehydrogenase from *Artemisia annua*.

In this study, *Saccharomyces cerevisiae* expressing the native *Artemisia annua* (*A. annua*) cytochrome P450 monooxygenase (CYP71AV1) and artemisinic aldehyde Δ11(13) reductase (DBR2) was used as a whole-cell biocatalyst to produce the immediate artemisinin precursor, dihydroartemisinic acid (DHAA). Although in vivo production of artemisinic acid (AA) has been reported, downstream conversion to DHAA has only been achieved by chemical conversion [15,16]. The biosynthesis of DHAA has been demonstrated, but productivity has yet to be optimized in a heterologous host [17]. CYP71AV1 is the critical enzyme that is capable of catalyzing three consecutive oxidation reactions at C-12 position of AD to AA (Figure 1) [18,19]. The heme catalytic site is situated at the C-terminal domain. Mutagenesis study has revealed the importance of C-terminal domain in determining the oxidizing activity [19]. Its electron supply partner is a flavoprotein and uses both flavin adenine dinucleotide (FAD) and flavin mononucleotide (FMN) as redox cofactors. In contrast to the previous report [7], our initial attempt to use CYP71AV1-overexpressing *E. coli* as the biocatalyst did not produce detectable levels of AA from extracellularly-fed AD; only a trace amount of artemisinin alcohol was detected. This may be due to the lack of internal membrane structures in *E. coli*, as P450 enzymes are known to be targeted to the endoplasmic reticulum. Therefore, we decided to test the use of yeast whole-cell biocatalysis. AA was successfully detected in the reaction medium, after CYP71AV1 activity was optimized through a series of enzyme engineering and transcriptional tuning processes. Kinetic investigation led us to identify the last oxidation step from artemisinic aldehyde (AO) to AA as the major bottleneck in the P450-mediated reaction. Introducing artemisinic aldehyde dehydrogenase (ALDH1) from *A. annua* resulted in a significant enhancement in AA titer. Subsequently, the co-expression of DBR2 and modulation of abiotic conditions resulted in a yield of ~230 mg/L DHAA production from 500 mg/L AD. Improvement in the percentage of conversion was achieved by in situ production of AD and DHAA from IPP and DMAPP by the addition of exogenous farnesyl pyrophosphate synthase (FPPS) and amorphadiene synthase (ADS) into the reaction. Approximately 80% conversion could be achieved from these initial building blocks. Since all the terpenoids were formed from the two precursors (IPP and DMAPP), our system presents a rapid and easy way to test and optimize the production of oxygenated terpenoids.

## 2. Results and Discussion

### 2.1. Enhancing CYP71AV1 Activity by Enzyme Engineering and Transcription Tuning

**Figure 2:**
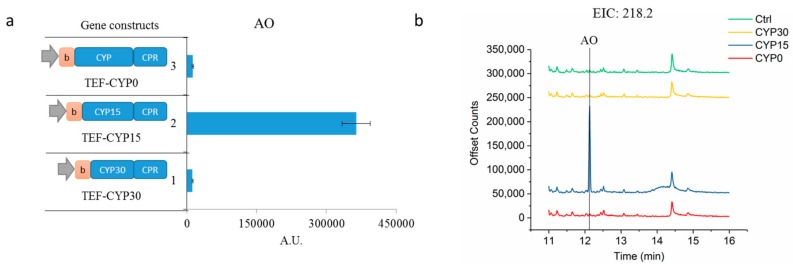

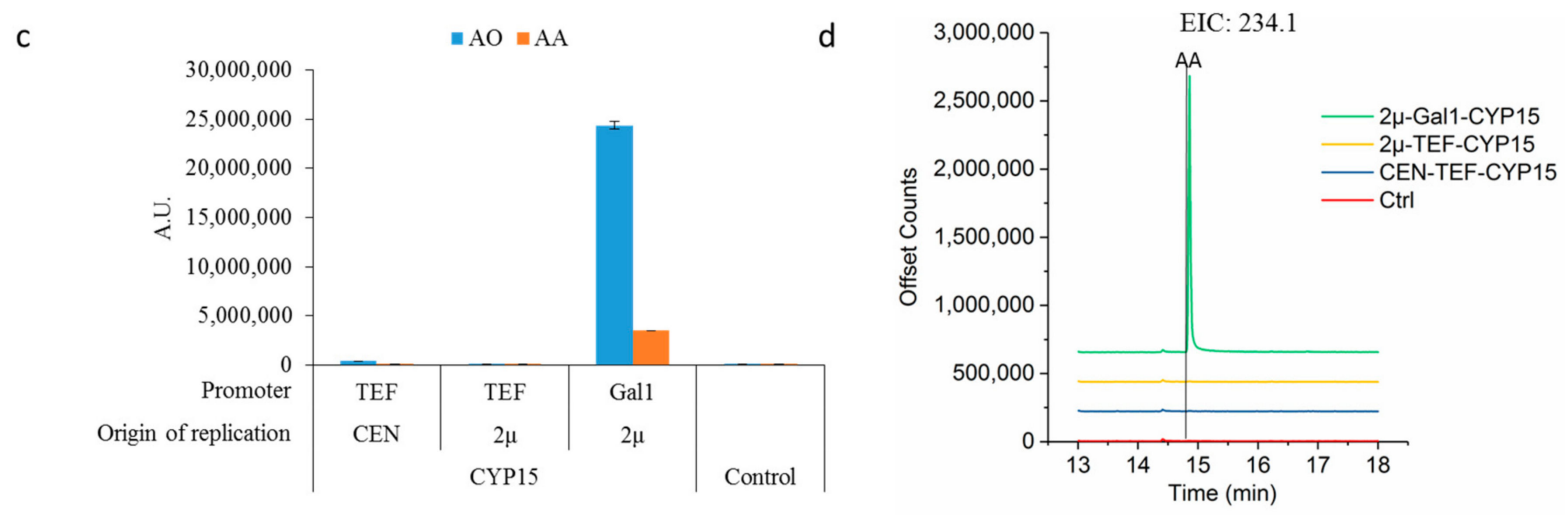
Optimization of P450 enzyme activity. (**a**) Evaluation the effect of N-terminal modification of CYP71AV1. Oxidized armorphadiene, namely artemisinic aldehyde, is only detected in the reaction with yeast overexpressing TEF-CYP15; (**b**) The gas chromatogram of extracted ion at *m*/*z* 218.2, which is the molecular ion of artemisinic aldehyde. The control is using the yeast cells with an empty vector; (**c**) Transcriptional tuning of CYP71AV1 expression and its effect on oxidized armorphadiene production. Artemisinic acid was detected in reaction with yeast overexpression pYES-Gal1-CYP15; (**d**) Gas chromatogram of extracted ion at *m*/*z* 234.1, which is the molecular ion of artemisinic acid. The control is using the yeast cell with an empty vector. All the measurements were done in triplicate, and their standard deviations are shown.

The first step in functionalizing AD is through regio-selective oxidation by the native *A. annua* cytochrome P450 monooxygenase (CYP71AV1) and its electron supply partner cytochrome P450 reductase (CPR). To mimic the highly effective P450 BM3 [20], we generated a fusion protein of CPR and CYP71AV1. However, hardly any oxidized products were detected (TEF-CYP0, Figure 2a,b), suggesting that the enzyme activity was limiting. Eukaryotic P450s are notoriously difficult to functionally express in heterologous hosts, partly because it is a membrane anchored protein. Engineering efforts to modify the N-terminal membrane anchoring sequence have been attempted [21]. Bioinformatics analysis predicted the first 29 amino acids of CYP71AV1 to be a membrane targeting sequence (MTS). It has been shown that truncating the MTS and replacing it with the more hydrophilic eight-residue peptide from bovine (8RP) would enhance P450 expression [22]. Hence, we systematically investigated the effect of N-terminus modification by removing 15 amino acids sequentially, and simultaneously incorporating the 8RP (Figure 2a). Effectively, the intermediate, artemisinic aldehyde (AO), was produced by the enzyme with the truncated first 15 amino acids (TEF-CYP15, Figure 2a,b). Unexpectedly, further truncations resulted in the loss of enzymatic activity (TEF-CYP30, Figure 2a,b), indicating that these residues may be involved in enzymatic activity. With this modification, AA was not detected in any of the whole cell reactions, suggesting that the CYP15 activity was still not sufficient. To further increase the amount of active enzymes, transcription was tuned by using a high-copy plasmid (pYES260, supplementary Table S1), and the strong inducible GAL1 promoter. As a result, AA was successfully detected in the yeast reaction after incubating AD with yeast whole cell for 8 h (2µ-Gal1-CYP15, Figure 2c,d).

### 2.2. Optimizing Artemisinic Acid Production by Host Screening and Co-Factor Engineering

**Figure 3:**
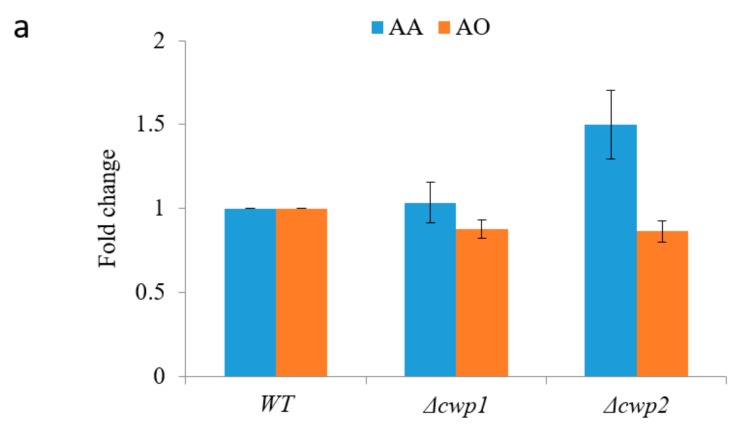

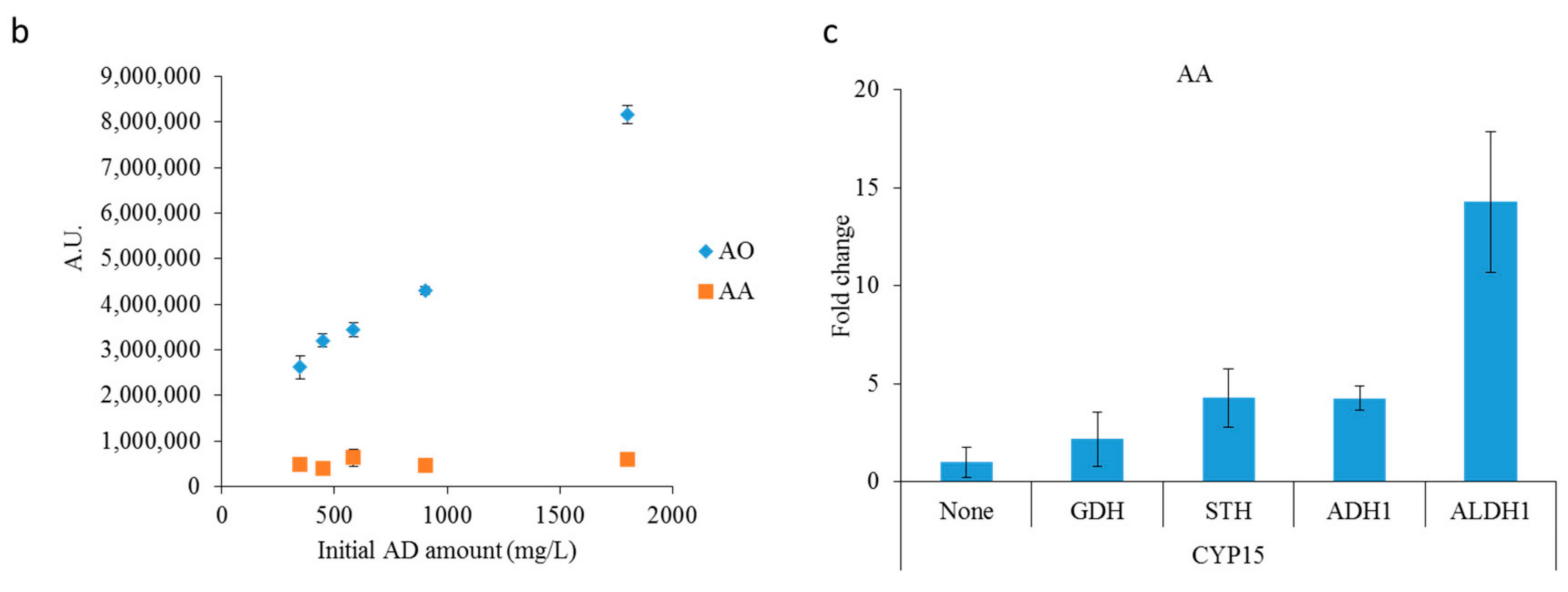
Optimization of artemisinic acid production. (**a**) Evaluation of the production of AA and AO by different yeast strains. The genotype is described in supplementary Table S1; (**b**) The initial rate of reaction of CYP15 was measured by changing the initial concentration of AD from 300 mg/L to 1800 mg/L. The reaction was allowed to be carried out at 28 °C for 30 min, and the oxygenated product levels were measured; (**c**) Evaluation of the effect of NAD(P)H-recycling systems for AA production. The fold change in DHAA production was calculated by the ratio between the production with and without NAD(P)H-recycling. All the measurements were done in triplicate, and the standard deviations are shown.

The majority of the metabolic intermediates were detected in the extracellular medium. We hypothesized that by permeabilizing or weakening the yeast cell wall, the mass transfer rate might be enhanced. Initially, we screened two different yeast phenotypes, each with a single gene deletion (∆cwp1, ∆cwp2), relating to cell wall structures (Supplementary Table S1). Growth rates were unaffected by these gene deletions. The cells were collected during the mid-exponential growth phase, and used for whole-cell biotransformation. As shown in Figure 3a, cwp2 deletion led to ~50% increase in AA production, as compared to the wild type. Concomitantly, AO was slightly decreased, suggesting that more intermediate was oxidized to the final product (Figure 3a). There was no significant improvement in AA production when cwp1 was deleted. Consistent with the observation that significant yeast cell wall thinning occurred with the deletion of cwp2 but not cwp1 [23], the higher conversion towards AA production with cwp2 deleted strain may be due to the increase in permeability of the precursor.

Next, we investigated the catalytic property of the whole-cell biocatalysis by examining the initial rate of reaction. Different amounts of AD were added to initiate the whole cell reaction. The reaction was rapidly terminated with 2 M hydrochloric acid at 30 min. The oxygenated products were extracted with ethyl acetate and subjected to GCMS analysis. Notably, the intermediate, AO, accumulated proportionally to the amount of AD added. However, AA production did not change significantly (Figure 3b), indicating that the last oxidation step was the main rate-limiting step. We hypothesized that it could be due to the limited co-factor supply, as three NADPH molecules are required to drive the oxidation reaction. To test this hypothesis, the commonly used NADPH recycling systems [24], namely, glucose dehydrogenase from *Bacillus megaterium* (GDH, EC 1.1.1.47) and transhydrogenase from *Escherichia coli* (STH, EC 1.6.1.1), were co-expressed with CYP15. The AA titer was indeed enhanced ~2 and ~4-fold with the respective enzymes (Figure 3c). We then tested the effects of native *A. annua* alcohol dehydrogenase (ADH1) and aldehyde dehydrogenase (ALDH1) [15,25]. There are two possible advantages: firstly, the enzymes may drive the desired flux towards AA; secondly, one molecule of NAD(P)H can be regenerated (Figure 1). As speculated, both dehydrogenase enzymes improved AA production. Remarkably, co-expressing ALDH1 resulted in a significant 14-fold enhancement in AA production (Figure 3c), and the level of AO was insignificant in this condition. This observation was consistent with previous reports [15,25], and further supported the proposal that the last oxidation step by CYP71AV1 was rate-limiting in the whole cell biocatalysis.

### 2.3. Dihydroartemisinic Acid Production and Reaction Condition Optimization

**Figure 4:**
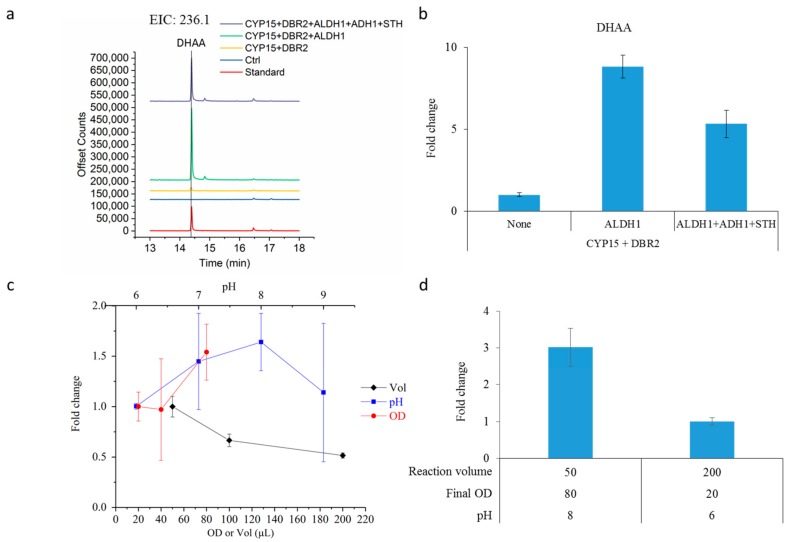
Production and optimization of dihydroartemisinic acid production. (**a**) Gas chromatogram of extracted ion at *m*/*z* 236.1, which is the molecular ion of DHAA. The standard was extracted from *Artemisia annua*. The control reaction used yeast cells grown in glucose medium; (**b**) Evaluation of the effect of NAD(P)H-recycling enzymes on DHAA production. The fold change in DHAA production was calculated by the ratio between the production with and without NAD(P)H-recycling. The strain overexpressing CYP15, DBR2 and ALDH1 was identified to be the optimal strain for DHAA production; (**c**) Optimization of the whole cell reaction conditions. Fold change in DHAA production was shown; (**d**) Fold change in DHAA production under the optimal and least optimal reaction conditions tested. All the measurements were done in triplicate, and the standard deviations are shown.

As DHAA is the immediate precursor of artemisinin [16,25,26], we then seek enzymatic route to produce DHAA directly from AD. DBR2 was previously identified as an aldehyde reductase that can reduce AO to dihydroartemisinic aldehyde (DHAO, Figure 1) [17]. We then codon optimized and overexpressed DBR2 in the CYP15-expressing yeast strain. A total ion chromatography (TIC) peak with the same retention time as the natural DHAA purified from *A. annua* was detected in this strain (CYP15-DBR2, Figure 4a). This observation indicated that the engineered CYP71AV1 and CPR fusion enzyme was able to oxidize DHAO to DHAA. Furthermore, the co-expression of ALDH1 significantly improved DHAA yield by 8-fold (Figure 4b). The yeast strain, with the overexpressions of CYP15, DBR2 and ALDH1, was termed CCDA. However, ADH1 and STH co-expression in the CCDA yeast whole-cell biocatalysis system resulted in a slight decrease in DHAA yield; the reason for this is yet to be unraveled. Hence, CCDA was further used to improve DHAA production.

**Figure 5:**
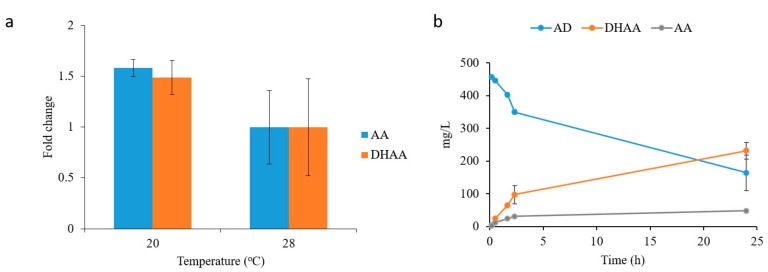
Optimization of dihydroartemisinic acid production. (**a**) Evaluation of the effect of the yeast growth temperature on DHAA and AA production; (**b**) Time course of yeast whole-cell biosynthesis of DHAA and AA from AD. All the measurements were done in triplicate, and the standard deviations are shown.

We next examined the contributions of three physical conditions that may affect the enzyme reactions. Firstly, the amount of functional catalysts that could be varied by tuning the final cell number in the reaction mixture; secondly, the reaction volume which is critical to affect the oxygen content; and thirdly, the pH of reaction, which would affect the chemical property and enzyme activity. Therefore, we used a univariant analysis method to determine the effect of each physical condition. As expected, when more biocatalysts were added to the reaction (from OD 20 to OD 80), DHAA yield was increased by 50% (Figure 4c). When the reaction volume was increased (from 50 to 200 µL), however, product yield was decreased (Figure 4c). The optimal reaction pH was found to be pH 8 (Figure 4c). When comparing the optimal condition (80 OD yeast cells in 50 µL reaction at pH 8) with the least optimal condition (20 OD yeast cells in 200 µL reaction at pH 6) examined, approximately 3-fold improvement in DHAA yield was produced (Figure 4d), indicating that the effects of conditions were additive. Furthermore, by growing the yeast cells at 20 *°*C in galactose medium, DHAA titer was further increased by 50% (Figure 5a). Hence, with the cumulative series of optimization, ~230 mg/L of DHAA accompanied with ~50 mg/L AA were produced from 500 mg/L AD. This titer was significantly greater than what was reported previously [17].

### 2.4. In Situ Production of Amorphadiene and Dihydroartemisinic Acid

**Figure 6:**
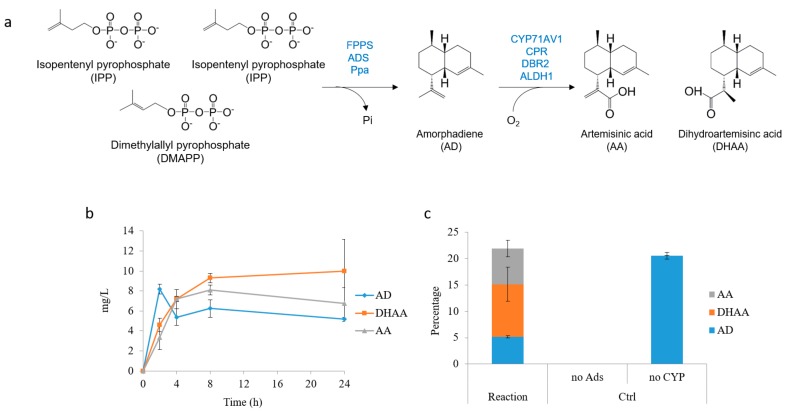
In situ cell-free biosynthesis of AD and DHAA. (**a**) Schematic representation of the production of AD, AA and DHAA from the building blocks IPP and DMAPP; (**b**) Time course of the production of AD, AA and DHAA; (**c**) The product distribution by the cell-free reactions. Two control reactions were used. The one without ADS, no amorphadiene was produced at all. The other without CYP15, no oxidized armorphadiene was detected. All the measurements were done in triplicate, and the standard deviations are shown.

Although the yield of DHAA was high, approximately 40% of AD remained unconverted to DHAA. This was likely to be due to the evaporation of AD into the head space of the reaction mixture when using such vessels [27,28]. In order to circumvent this challenge, one approach was to use an organic layer (10% (*v*/*v*) dodecane) to trap the volatile AD produced. Unfortunately, dodecane was found to quench the reaction (results not shown). Another strategy was to produce AD and DHAA in situ from the isoprenoid building blocks, namely IPP and DMAPP (Figure 6a). The substrates are water soluble, and in vitro multienzyme biosynthesis has been optimized [29]. Coincidentally, in vitro AD biosynthesis is also optimal at pH 8, which is compatible with the yeast whole-cell reaction. As a proof-of-concept, we set up three different reactions: one with complete reconstituted pathway enzymes comprising extracellularly added FPPS, ADS, inorganic pyrophosphatase (Ppa) [30] and the CCDA yeast strain grown in galactose medium; one control reaction with all the pathway constituents except ADS; and another control reaction with yeast cells grown in glucose medium (no CYP). All three reactions were initiated by adding IPP (~200 µM) and DMAPP (~100 µM) to a buffered solution (50 µL, 10 mM MgCl_2_, 100 mM Tris-HCL, pH 8). The extent of the reaction was monitored at 2, 4, 8 and 24 h at 28 °C. At each time point, the reaction was terminated with 2 M HCL, and the products were extracted with equal volumes of ethyl acetate. As shown in Figure 6b, the reaction was almost completed at 8 h. Approximately 20 mg/L AD and oxygenated AD were produced, which is >95% conversion from IPP and DMAPP. Similar titer of AD was produced in the control reaction without CYP (Figure 6c). Moreover, the percentage of AD converted to acid was increased to ~80%. Future studies will involve the balancing of the rates of production and consumption of AD so as to further optimize the biosynthesis in vitro.

Recently, in vitro multienzyme synthesis of terpene backbones, such as limonene and amorpha-4,11-diene, has achieved an industrially relevant titer from cheap carbon sources [30,31,32,33]. The study herein demonstrated further oxygenation of terpene backbones by a combination of whole cell catalysts and controlled reaction media conditions in a one-pot reaction. This approach is scalable, and is appealing for the further development of economically and ecologically beneficial downstream processes [34].

## 3. Materials and Methods

### 3.1. Strains and Plasmids

The microbial strains and plasmids used in this study were summarized in supplementary Table S1. The pathway genes, *cyp71av1*, *cpr*, *aldh1*, *adh1* and *dbr2*, were codon optimized and synthesized by Genescript, and they were cloned into modified p416-TEF (ATCC 87368) and pYES260 (EUROSCARF, Bad Homburg, Germany) vector by CLIVA method [35]. The primers used are listed in supplementary Table S2. The constructed plasmids were transformed into and amplified in *E. coli* DH10B strain. Then the purified plasmid was transformed into yeast strain with the lithium acetate mediated method [36]. All the yeast shuttle vectors carried the Ura3 auxotrophic marker and can be selected by synthetic media lacking uracil. The colonies were formed after incubating at 30 °C for 2–3 days. Then, colony PCR was carried out to confirm the presence of the plasmid. The correct colony was then grown in synthetic medium lacking uracil for whole cell biocatalysis.

### 3.2. Yeast Growth and Protein Expression

1 mL culture was grown overnight in 2% glucose synthetic medium lacking uracil (20 g/L glucose, 6.7 g/L drop-out base (USbiological, Salem, MA, USA), 2 g/L drop-out mix without uracil (USbiological, Salem, MA, USA)), until stationary phase at 28 °C, for 300 rpm in a shaking incubator. The yeast growth was monitored at an optical density of 600 nm. Cells harboring p416-TEF plasmid were inoculated again in 10 mL 2% glucose synthetic medium. For cells harboring pYES260 plasmid, enzyme expression was induced by inoculating the cells, to final OD_600_ 0.05, in 1.8% galactose synthetic medium (2 g/L glucose, 18 g/L Galactose, 6.7 g/L drop-out base (USbiological, Salem, MA, USA), 2 g/L drop-out mix without uracil (USbiological, Salem, MA, USA)). Subsequently, the cells were grown at 28 °C or 20 °C till OD_600_ reaching approximately 4, and collected for whole-cell biocatalysis.

### 3.3. Amorpha-4,11-Diene Purification

AD was produced via the non-mevalonate pathway enzymes and amorphadiene synthase (ADS) in MG1655(DE3) strain, as previously described [10]. The strain was grown in 100 mL 2xPY (10 g/L yeast extract, 20 g/L peptone, 10 g/L sodium chloride, pH 7) medium supplemented with 10 g/L glucose for 2 days, at 28 °C. The production was induced with 0.1 mM isopropyl β-d-1-thiogalactopyranoside (IPTG), when the OD_600_ reached 0.6. 2 g C18 beads were added simultaneously to capture the AD produced. 24 h after induction, the beads were allowed to sediment, and the bacterial culture was carefully removed. Then, the beads were resuspended in deionized water, and loaded into a chromatography column (Bio-Rad, Hercules, CA, USA). The beads were washed twice with deionized water to remove any residual bacterial cells. Then, the AD was eluted with hexane (~1 mL) three times. GCMS analysis was carried out to determine the extraction efficiency. Approximately 50% of the AD produced could be eluted with hexane. The hexane was further evaporated by rotary vacuum concentrator, and the AD was dissolved in ethanol for yeast whole cell biocatalysis. The concentration of AD was determined by comparing with a known amount of caryophyllene by GCMS.

### 3.4. Overexpression and Purification of FPPS, ADS and Ppa

Overexpression and purification of FPPS, ADS and Ppa were performed as described [29], via the immobilized metal affinity chromatography method [37]. The enzyme concentrations were determined by Micro BCA protein assay kit (Thermo Scientific, Waltham, MA, USA). Sodium dodecyl sulfate-12% polyacrylamide gel electrophoresis was carried out, and the protein was judged to be at least 90% pure before it was used for biocatalysis (Supplementary Figure S4). The enzyme concentrations used were according to the optimal concentrations determined before [30]; 9 ng/µL FPPS, 200 ng/µL ADS, and 50 ng/µL Ppa were added to 50 µL buffered yeast reaction mixture with 100 µM DMAPP, 200 µM IPP and 10 mM magnesium chloride.

### 3.5. Yeast Whole Cell Biocatalysis and Product Extraction

Approximately 2 OD* mL of yeast cells were collected and mixed with AD in a 100 µL reaction, or as otherwise stated in the text, containing 100 mM Tris-HCL at the specified pH. The reaction was carried out in a 2 mL GC vial, sealed with screwed cap to prevent evaporation of AD. At the end of reaction, 10% 2 M HCL, and 100 µL of ethyl acetate containing 50 mg/L caryophyllene was added to extract AD and its oxidized products. After vortexing at room temperature for 10 min, the organic layer was separated from the aqueous solution by centrifugation at 15,000× *g* for 10 min. Then it was further diluted either 2 or 50 times for GCMS measurement.

### 3.6. GCMS Analysis of AD, AOH, AO and AA

1 μL of the sample was injected into an HP-5 column (Agilent Technologies 7890A gas chromatograph-mass spectrometry, Agilent, Santa Clara, CA, USA). The gas chromatography oven program was set according to previous study [7]. In brief, it was initially held at 100 °C for 5 min, and then heated 100–150 °C at 30 °C/min, 150–205 °C at 5 °C/min, 205–300 °C at 50°C/min, and held at 300 °C for another minute. Scan mode was used to detect the compounds. With that temperature profile, amorphadiene (AD), artemisinic alcohol (AOH), artemisinic aldehyde (AO), artemisinic acid (AA) and dihydroartemisinic acid (DHAA) were eluted at 9.3 min, 11.9 min, 12.1 min, 14.8 min and 14.4 min, respectively. The peak area was calculated with the software provided by the manufacturer. AD concentrations were pre-determined relative to the internal standard, trans-caryophyllene, with known concentrations. DHAA concentration was calculated against the external authentic standard extracted from *A. annua*. AA concentration was estimated based on the DHAA concentration calculated.

## Supplementary Materials

The following are available online. Figure S1: The mass spectrometry of the artemisinic alcohol, artemisinic aldehyde, artemisinic acid, and dihydroartemisinic acid. Figure S2: Vector map of pYES-Gal1-CYP15-Gal10-DBR2-T2A-ALDH1 and pYES-Gal1-CYP15-Gal10-DBR2-T2A-ALDH1-Gal1-ADH1-P2A-STH. Figure S3: SDS PAGE gel analysis of the purified FPPS, ADS, and Ppa. Table S1: The yeast strains and plasmids used in the study. Table S2: The CLIVA primers used to construct the plasmids in the study.
