# Supplementary material for: Multienzyme Biosynthesis of Dihydroartemisinic Acid
Source: Molecules. 2017 Aug 28;22(9):1422. doi: 10.3390/molecules22091422 (PMC6151439; doi:10.3390/molecules22091422)
Supplement: Supplementary file 1 [file molecules-22-01422-s001.pdf]

Supporting Information

Supplementary figure S1. The mass spectrometry of the artemisinic alcohol, artemisinic aldehyde, artemisinic acid, and dihydroartemisinic acid.

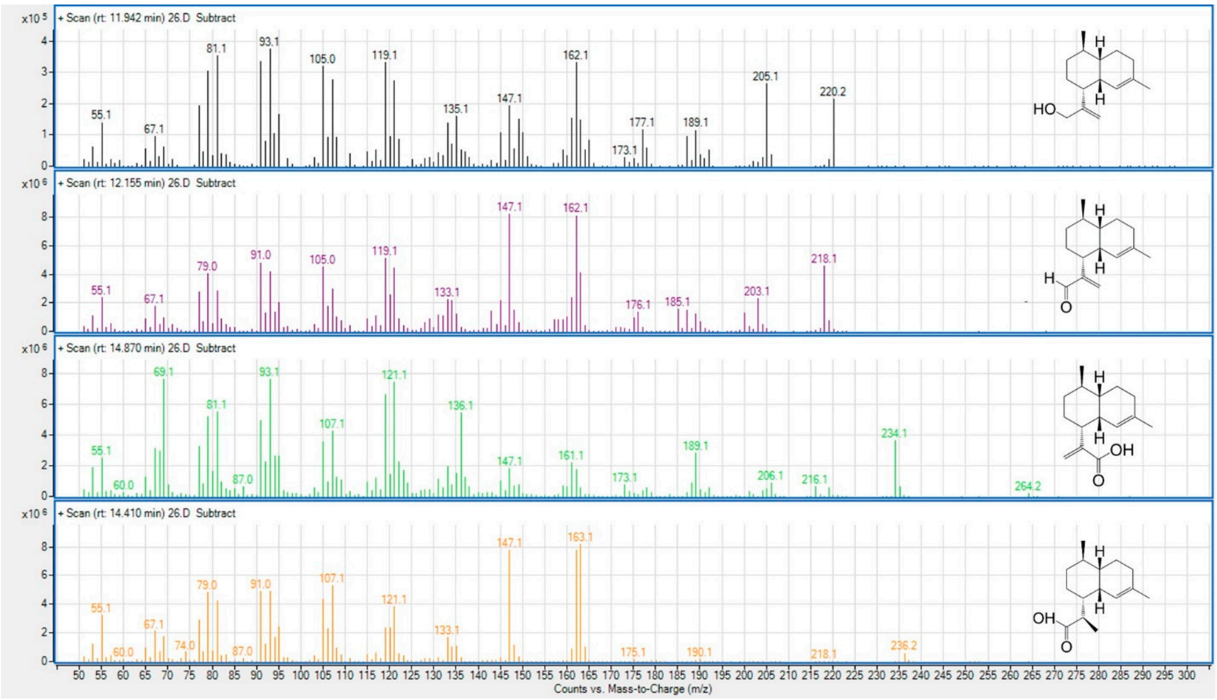

Supplementary figure S2. Vector map of pYES-Gal1-CYP15-Gal10-DBR2-T2A-ALDH1 and pYES-Gal1-CYP15-Gal10-DBR2-T2A-ALDH1-Gal1-ADH1-P2A-STH

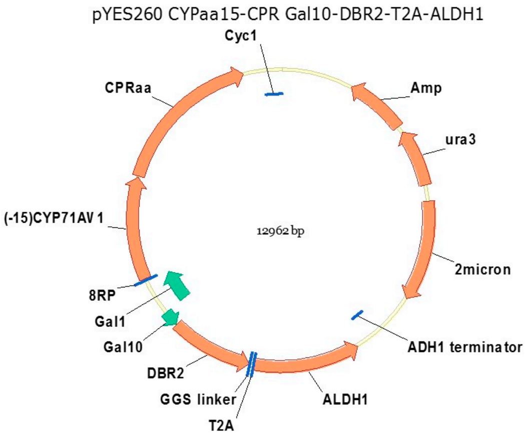

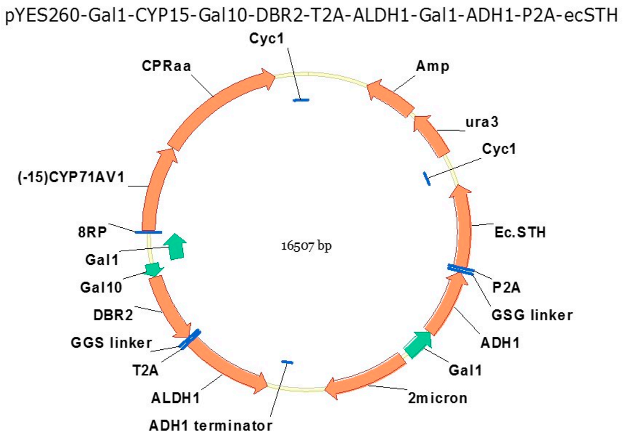

Supplementary figure S3. SDS PAGE gel analysis of the purified FPPS, ADS, and Ppa. The abbreviations are: T, total soluble protein; B, binding supernatant; P, purified enzyme. The molecular weight of protein ladder was shown.

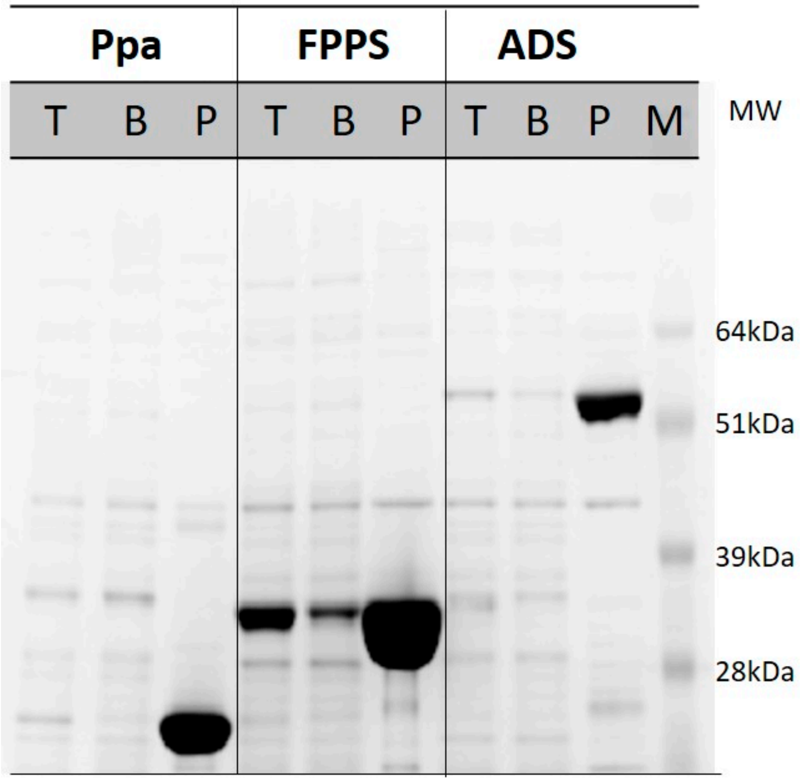

Supplementary table S1. The yeast strains and plasmids used in the study.

Strain	Genotype	Source
BY4741	MATa; his3Δ 1; leu2Δ 0; met15Δ 0; ura3Δ 0	EUROSCARF, Germany
dCWP1	BY4741; Mata; his3D1; leu2D0; met15D0; ura3D0; YKL096w::kanMX4	EUROSCARF, Germany
dCWP2	BY4741; Mata; his3D1; leu2D0; met15D0; ura3D0; YKL096w-a::kanMX4	EUROSCARF, Germany
**Plasmids**	**Properties**	
p416-TEF	CEN *ori*; TEF promoter; URA3; pBR322 *ori*; AmpR	ATCC 87368
pYES260	2µ *ori*; Gal1 promoter; URA3; pBR322 *ori*; AmpR	EUROSCARF, Germany
p416-TEF	p416 *ori*; TEF promoter; URA3; pBR322 *ori*; AmpR; CYP0 overexpression	This study
P416-TEF-CYP15	p416 *ori*; TEF promoter; URA3; pBR322 *ori*; AmpR; CYP15 overexpression	This study
P416-TEF-CYP30	p416 *ori*; TEF promoter; URA3; pBR322 *ori*; AmpR; CYP30 overexpression	This study
pYES260-TEF-CYP15	pYES260 *ori*; TEF promoter; URA3; pBR322 *ori*; AmpR; CYP15 overexpression	This study
pYES260-Gal1-CYP15	pYES260 *ori*; Gal1 promoter; URA3; pBR322 *ori*; AmpR; CYP15 overexpression	This study
pYES260-Gal1-CYP15-Gal1-GDH	pYES260 *ori*; Gal1 promoter; URA3; pBR322 *ori*; AmpR; CYP15 and GDH overexpression	This study
pYES260-Gal1-CYP15-Gal1-STH	pYES260 *ori*; Gal1 promoter; URA3; pBR322 *ori*; AmpR; CYP15 and STH overexpression	This study
pYES260-Gal1-CYP15-Gal1-ADH1	pYES260 *ori*; Gal1 promoter; URA3; pBR322 *ori*; AmpR; CYP15 and ADH1 overexpression	This study
pYES260-Gal1-CYP15-Gal1-ALDH1	pYES260 *ori*; Gal1 promoter; URA3; pBR322 *ori*; AmpR; CYP15 and ALDH1 overexpression	This study

pYES260-Gal1-CYP15-Gal10-DBR2	pYES260 *ori*; bidirectional Gal1 promoter; Gal10 promoter; URA3; pBR322 *ori*; AmpR; CYP15 and DBR2 overexpression	This study
pYES260-Gal1-CYP15-Gal10-DBR2-T2A-ALDH1*	pYES260 *ori*; bidirectional Gal1 promoter; Gal10 promoter; URA3; pBR322 *ori*; AmpR; CYP15 overexpression, DBR2 and ALDH1 overexpression via T2A system	This study
pYES260-Gal1-CYP15-Gal10-DBR2-T2A-ALDH1-Gal1-ADH1-P2A-STH*	pYES260 *ori*; bidirectional Gal1 promoter; Gal10 promoter; URA3; pBR322 *ori*; AmpR; CYP15 overexpression, DBR2 and ALDH1 overexpression via T2A system, ADH1 and STH overexpression via P2A system	This study

* Please refer to supplementary figure S2 for details

Supplementary table S2. The CLIVA primers used to construct the plasmids in the study.

Primer name	Sequence (5’→ 3’)
I-TEF(CYPf)_r	TATTTAATC*GAAACTTAG*ATTAGATTGCTATGCTTT
I-CYP(TEFr)_f	CTAAGTTTC*GATTAAATA*AGGAGGAATAACAT
I-TEF(CYPr)_f	TAAAACAGA*TCATGTAAT*TAGTTATGTCACGCTT
I-CYP(TEFf)_r	ATTACATGAT*CTGTTTTAT*CAGACCGCTTCT
I-CYP15(BOVr)_f	CAGTTTT*TATTGCG*CTGGCGA
I-Bov(CYP15)_r	CGCAATA*AAAACTG*CTAATAACAGAGC
I-CYP30(BOVr)_f	GCAGTTT*TTCGTAGC*AAGAGCACCA
I-Bov(CYP30)_r	GCTACGAA*AAACTGC*TAATAACAGAGC
I-pYES(TEFf)_r	CTATAGT*GGATCATC*CCCAC
I-TEF(pYESr)_f	GATGATCC*ACTATAG*CTTCAAAATGTTTCTAC
I-TEF(CYPf)_r	TAATCGAA*ACTTAGA*TTAGATTGCTATGC
I-CYP(TEF)_f	TCTAAGTT*TCGATTA*AATAAGGAGGAAT
I-pYES_CYC1TT(CPRTT)_r	CGAAGAGG*ACGGTTCC*TGGGCTTTTGC
I-CPR_CYC1TT(YESTT)_r	GGAACCGT*CCTCTTCG*CTATTACGCCAG
I-pYES_AMP(TEFCYPf)_f	GTGAGGTAAC*CTGGCGT*TTTTCCATAGGCT
I-TEF_CYP(YESAMPf)_f	ACGCCAG*GTTACCTCAC*TCATTAGGCACCC
I-pYESTT(-)-f	CGAGAATCT*TTATTTTCAG*GG
I-pYESPro(-)-r	CTTAATATTC*CCTATAGTGAGT*CGT
I-DBR2(pYESf)-r	CTGAAAATA*AAGATTCTCG*TTACAGCAGGCTGCCCTT
I-DBR2(pYESr)-f	ACTCACTATAG*GGAATATTAAG*ATGTCCGAGAAACCTACCC
I-pETK(pYESf)-r	CTGAAAATA*AAGATTCTCG*TCCTTTCGGGCTTTGTTAG
I-ADH1(pYESr)-f	ACTCACTATAG*GGAATATTAAG*AATGGCACAGAAAGCCC
I-ALDH1(pYESr)-f	ACTCACTATAG*GGAATATTAAG*AATGAGCAGCGGTGCC
I-GDH(pYESr)-f	ACTCACTATAG*GGAATATTAAG*ATGCATCACCATCACCATC
I-STH(gal1r)_f	GAATGC*CACATTC*CTACGATTAC
I-gal1(STH)_r	GAATGTG*GCATTC*TTAATATTCCCTATAGTG
I-Gal10_DA(GAL1f)_f	TCCGTA*CTTCAA*TATAGC*AATGAGCAGT

I-GAL1(GAL10f)_f	GCTATA*TTGAAG*TACGGA*TTAGAAGCCG
I-GAL1(ADHtr)_r	GGCAT*GCAGT*GGATC*ATCCCCACG
I-Gal10_DA-ADHtt(Gal1r)_r	GATCC*ACTGC*ATGCC*GGTAGAGGT

*indicated the phosphorothioate modification
